# The Role of Blood Inflammatory Biomarkers and Perineural and Lympho-Vascular Invasion to Detect Occult Neck Lymph Node Metastases in Early-Stage (T1-T2/N0) Oral Cavity Carcinomas

**DOI:** 10.3390/cancers17081305

**Published:** 2025-04-12

**Authors:** Stefania Troise, Fabio Di Blasi, Maria Esposito, Giulia Togo, Daniela Pacella, Raffaele Merola, Rosa Maria Di Crescenzo, Stefania Staibano, Vincenzo Abbate, Paola Bonavolontà, Giovanni Salzano, Riccardo Nocini, Carlos Navarro Cuellar, Giovanni Dell’Aversana Orabona

**Affiliations:** 1 Maxillofacial Surgery Unit, Department of Neurosciences, Reproductive and Odontostomatological Sciences, University of Naples Federico II, 80131 Naples, Italy; fabio93di@gmail.com (F.D.B.); meryesposito1310@gmail.com (M.E.); vincenzo.abbate@unina.it (V.A.); paola.bonavolonta@unina.it (P.B.); giovannisalzanomd@gmail.com (G.S.); giovanni.dellaversanaorabona@unina.it (G.D.O.); 2 Maxillofacial and ENT Surgery Unit, Tumors National Institute IRCCS G. Pascale, 80131 Naples, Italy; giulia.togo@gmail.com; 3 Department of Public Health, University of Naples Federico II, Via Sergio Pansini 5, 80131 Naples, Italy; daniela.pacella@unina.it; 4 Anesthesia and Intensive Care Medicine, Department of Neurosciences, Reproductive and Odontostomatological Sciences, University of Naples Federico II, 80131 Naples, Italy; rafmerola2003@gmail.com; 5 Pathology Unit, Department of Advanced Biomedical Sciences, University of Naples Federico II, 80131 Naples, Italy; rosamariadicrescenzo@gmail.com (R.M.D.C.); stefania.staibano@unina.it (S.S.); 6 Ear, Nose and Throat, Department of Surgical Sciences, Dentistry, Gynaecology and Paediatrics, University of Verona, 37129 Verona, Italy; riccardo.nocini@gmail.com; 7 Division of Oral and Maxillofacial Surgery, Hospital General Universitario Gregorio Marañon, Universidad Complutense de Madrid, 28040 Madrid, Spain; canava03@ucm.es

**Keywords:** oral cavity carcinoma, occult neck metastases, inflammatory biomarkers, perineural invasion, lympho-vascular invasion

## Abstract

Background/Objectives: Oral cavity carcinomas (OCCs) represent roughly 50% of all head and neck cancers. The risk of occult neck metastases for early-stage OCCs ranges from 15 to 35%, thus the need to develop tools that can support the diagnosis detecting these neck metastases. Inflammatory biomarkers and perineural and lympho-vascular invasion are emerging as effective in this field. The aim of this study is to demonstrate the effectiveness of these parameters to detect occult neck metastases in early-stage (T1-T2/N0) OCCs. Methods: A retrospective analysis was conducted on 81 patients surgically treated for early-stage OCC. For all patients, data regarding TNM, pN status after the histopathological examination, inflammatory biomarkers, and perineural and lympho-vascular invasion have been obtained. A statistical analysis was performed using the receiver operating characteristic (ROC) curve to calculate the optimal cutoff values for SII, SIRI, PLR, and NLR. Results: Fifty-eight patients confirmed N0 status after surgery, while twenty-three resulted pN+. The best cut-off to detect occult neck metastases were PLR 249.30, NLR 13.10, MLR 0.439, SII 1043.12, and SIRI 1.85. The accuracy to detect occult neck metastases was PLR 75%, NLR 81%, MLR 74%, SII 73%, SIRI 70%, perineural invasion 70%, and lympho-vascular invasion 83%. Conclusions: Our results confirm that inflammatory biomarkers and perineural and lympho-vascular invasion are effective in detecting occult neck metastases in early-stage OCCs. The clinical relevance of this study is that these parameters could be used routinely as preoperative tools to support diagnosis and to help surgeons in the decision-making process, particularly regarding surgical indications for neck lymph nodes treatment.

## Simple Summary

Managing oral cavity carcinomas remains a challenge for clinicians, with substantial direct and indirect costs associated with diagnosis and treatment. Therefore, developing a fast and cost-effective prognostic system to identify high-risk patients is essential. Inflammatory biomarkers such as the systemic immune-inflammation index (SII), systemic inflammation response index (SIRI), platelet-to-lymphocyte ratio (PLR), and neutrophil-to-lymphocyte ratio (NLR) have been widely recognized in oncology for their prognostic value, given their accessibility and low cost. Inflammatory biomarkers and perineural and lympho-vascular invasion are emerging as effective tools in this field. This study aimed to demonstrate the effectiveness of these parameters to detect occult neck metastases in early-stage (T1-T2/N0) OCCs.

## 1. Introduction

Oral cavity carcinomas (OCCs), constituting about half of all head and neck carcinomas [1], pose a substantial global health concern, ranking as the sixth most common neoplasm globally. It includes a diverse range of malignancies originating in the lips, tongue, floor of the oral cavity, palate, gingiva, and buccal mucosa, and between 25% and 45% of cases can metastasize to cervical lymph nodes, according to staging, grading, and anatomical site [1,2,3]. The staging of oral cavity carcinomas plays a central role in assessing the disease’s extent and severity, influencing treatment choices and predicting prognosis. The widely embraced staging system for these carcinomas is the TNM (tumor, node, and metastasis) classification, with its latest edition released in 2017 (UICC/AJCC 8th Edition) [4]. At the time of diagnosis, only about 10% of patients presents clinical nodal metastasis [5]. In most cases, clinically, these metastases are undetected, with a rate of occult metastasis ranging from 15% to 34% [6,7].

Whereas the lymph node status represents one of the most important prognostic factors, the management of a clinically negative neck becomes an important challenge for the surgeon, especially in early-stage (T1–T2) oral cancers. A watchful waiting approach with close clinic-radiological surveillance can be adopted, otherwise elective neck dissection or biopsy of the sentinel lymph node have to be considered. It is crucial to highlight that elective neck dissection can cause complications such as shoulder dysfunction or myofascial pain and all the complications related to the second surgical site; moreover, in most of cases (about 70%), the neck dissection result is histologically negative, constituting an overtreatment [8].

Sentinel lymph node biopsy (SLNB) has become an important method for detecting metastatic spread in head and neck cancer. This approach focuses on locating and sampling the initial lymph node(s) responsible for draining the primary tumor site, enabling a precise pathological evaluation. In cases where metastases are found in the sentinel node, a neck dissection is required [9].

SLNB, however, presupposes the use of adequate instruments and qualified healthcare professionals, which are not always easily available in all the hospitals, hence the need to identify preoperative parameters that are easily accessible and reproducible in all healthcare structures.

There is a need of identifying laboratory, clinical, and imaging parameters capable of detecting occult metastases or at least minimizing the risk of their presence, ensuring the selection of the most appropriate treatment while avoiding both undertreatment and overtreatment, along with their associated morbidity. Different parameters are important to estimate the risk of neck metastases in oral squamous cell carcinoma (OSCC), such as T-stage, grading, tumor location, lymphovascular invasion (LVI), tumor budding, perineural invasion, depth of invasion (DOI), patient’s age, and inflammatory status, in particular the neutrophil-to-lymphocyte ratio (NLR) [9,10].

Lymphovascular invasion (LVI) is characterized by the presence of tumor cells within clearly defined spaces lined by endothelium, involving either blood vessels or lymphatic channels [11].

This pathological process involves tumor cells infiltrating endothelial-lined vascular or lymphatic spaces within the extracellular matrix, facilitating their spread to lymph nodes or distant sites [12]. LVI plays a crucial role in tumor dissemination through the lymphatic system and significantly contributes to the development of lymph node metastases (LNM), a key prognostic factor in patients with oral squamous cell carcinoma (OSCC). Approximately 25% to 45% of OSCC patients present with neck lymph node metastases [13].

The eighth edition of the AJCC staging system highlights the importance of documenting various histopathological features in OSCC, including LVI, as an additional prognostic marker. However, its effectiveness in accurately stratifying OSCC patients based on recurrence or survival risks remains an ongoing debate [14]. LVI positivity is related not only to an increased risk of regional lymph node metastases but also to a poorer prognosis concerning disease recurrence and overall survival [15,16].

Perineural invasion (PNI) refers to the process by which tumor cells infiltrate or grow along nerve fibers. It is generally diagnosed when tumor cells surround at least one-third (33%) of a nerve’s circumference, while cases with less involvement are considered to reflect tumor proximity rather than true invasion [17]. In oral cavity squamous cell carcinoma (OCSCC), PNI is a well-recognized indicator of poor prognosis. Its presence is associated with a higher risk of neck lymph node metastases and is linked to decreased disease-specific and overall survival rates, even in patients with early-stage tumors [18,19,20].

Furthermore, an elevated proinflammatory state has been identified as a potential marker for cancer progression and lymph node metastasis. The prognostic value of specific blood-derived inflammatory biomarkers—such as the neutrophil-to-lymphocyte ratio (NLR), platelet-to-lymphocyte ratio (PLR), systemic immune-inflammation index (SII), and systemic inflammation response index (SIRI)—has been extensively reported in various malignant neoplasms, including lung adenocarcinomas, early-stage clear-cell carcinomas, endometrioid and mucinous ovarian carcinomas, hepatocellular carcinomas, and salivary gland tumors [21,22,23,24].

The clinical relevance of these biomarkers lies in their accessibility and reliability, as they can be easily measured through routine blood counts in oncological patients, providing valuable diagnostic information [25,26].

The objective of this study is to demonstrate the effectiveness of these preoperative parameters, such as blood inflammatory biomarkers and histopathological features as lymphovascular invasion and perineural invasion, in detecting neck lymph node metastases in early-stage oral cavity carcinomas (T1-T2) with a clinically negative neck (N0), considered both individually and in combination. The clinical relevance of this study could be to routinely evaluate these parameters in the management of these tumors, to support the surgeon in the decision-making process, avoiding the risk of overtreatment/undertreatment.

## 2. Materials and Methods

### 2.1. Study Design

A retrospective analysis was conducted between January 2012 and May 2022 on patients admitted at the Maxillofacial Surgery Unit of the University Federico II of Naples for cT1-T2/cN0 OCCs. All the data were obtained by the retrospective analysis of patients’ medical records.

All the patients which satisfied these inclusion criteria were enrolled in the study:

- Diagnosis of squamous OCC confirmed by a preoperative incisional biopsy, evaluating perineural and lympho-vascular invasion;
- Classified early-stage cT1−T2 OCC with clinically negative neck (cN0), according to preoperative American Joint Committee on Cancer (AJCC) classification (8th edition) [4];
- No previous/simultaneous cancer at any other site and no previous radiotherapy or chemotherapy;
- Availability of preoperative blood exams, performed 7 days before surgery;
- Surgical indication to perform an elective neck dissection (END) following the last National Comprehensive Cancer Network guidelines (NCCN 2022), in particular a depth of invasion (DOI) > 3 mm [27];
- A minimum of 24 months of postoperative follow-up.

Patients who did not satisfy the inclusion criteria or met the following criteria were excluded from this study:

- Radiotherapy or chemotherapy in the clinical history;
- Previous/simultaneous tumor in other sites;
- Classified clinical advanced OCC (cT3-cT4);
- Clinically positive neck (cN+) or presence of distance metastasis (M+);
- Lost at follow-up.

The research adhered to the principles outlined in the Declaration of Helsinki, and informed consent was obtained from each involved patient for all diagnostic and surgical procedures. Given the retrospective and observational non-interventional nature, the study was waived for ethics committee approval.

### 2.2. Data Collection

The data were collected from the patients’ medical records and included demographic information (age, sex), tumor localization and subsequent surgical treatment, clinical classification of the tumor, and pathological stage according to the Union for International Cancer Control/American Joint Committee on Cancer (8th edition) [8]. The study included only patients classified with OCC in the early stage (T1/T2, cN0), who, according to current literature [20,21], were considered candidates for elective neck dissection (END), in particular with a DOI > 3 mm.

To acquire these data, each patient underwent a complete physical examination, neck ultrasonography (US) and magnetic resonance imaging (MRI) of the head and neck within 20 days before surgery. Each patient underwent a consistent incisional biopsy in order to be able to histologically analyze on the sample the DOI, the perineural invasion, and the lympho-vascular infiltration.

The diagnosis of early-stage carcinoma (T1-T2) was established after a complete staging evaluation using MRI, neck ultrasound, and total-body Positron Emission Tomography/Computer Tomography (PET/CT) by a multidisciplinary team composed of surgeons, oncologists, and radiologists. Once the early-stage carcinoma diagnosis was confirmed, blood samples were collected, and the patient was scheduled for surgery, which was performed within 7 days from the diagnosis. Given the short time frame between diagnosis and surgery, the blood test indicators accurately reflected the early-stage status of the disease.

The hematological parameters such as neutrophil count, lymphocyte count, platelet count, and monocyte count, were calculated on blood exams within one week before the surgery. The neutrophil-lymphocyte ratio (NLR) was defined as the neutrophil count divided by the lymphocyte count; the platelet–lymphocyte ratio (PLR) was obtained by dividing the platelet count by the lymphocyte count; the systemic immune-inflammation index (SII) was defined as the neutrophil count multiplied by the platelet count divided by the lymphocyte count; and the systemic inflammation response Index (SIRI) was defined as the neutrophil count multiplied by the monocyte count divided by the lymphocyte count.

Follow-up was performed with periodic 6-month outpatient checks, up to a minimum of 24 months, performing ultrasound and MRI every 6 months and an annual PET/CT scan.

### 2.3. Statistical Analysis

Data are summarized with mean and standard deviation for continuous variables and with absolute frequency and percentage for categorical variables. Student’s *t*-test was used to investigate the difference between means for continuous variables. Chi-square test was used to investigate difference between groups for categorical variables. Receiver operating characteristic (ROC) curves were constructed, and the corresponding areas under the curves (AUCs) were computed to evaluate the diagnostic performances of the NLR, PLR, MLR, SIRI, and SII markers in detecting the presence of metastasis. The gold standard for confirming the presence of lymph node metastases was postoperative histopathological examination. For each marker, optimal cut-offs were determined maximizing the Youden index and corresponding accuracy, sensitivity, specificity, positive predictive value (PPV), and negative predictive value (NPV) were provided. To calculate sensitivity and specificity, positivity was defined as having a marker value over the optimal threshold or having either perineural invasion or lympho-vascular invasion. Negativity was defined as having a marker value below the optimal threshold or not having perineural invasion or lympho-vascular invasion. True positivity and true negativity were then evaluated against the presence or absence of metastases, respectively, as provided by the gold standard (i.e., postoperative histopathological examination). Accuracy was then computed as the sum of true positives and true negatives divided by the total number of observations. Associations between the considered factors and the outcome of interest were evaluated using univariable logistic regression models. For all analyses, a *p* < 0.05 was considered significant. All analyses were performed using R statistical software, version 4.4.0.

## 3. Results

### 3.1. Patient Characteristics

Considering the inclusion and exclusion criteria, 81 patients were enrolled in the study. All the features of these included patients are shown in Table 1.

The following data present a distribution of tumors across various anatomical regions within the oral cavity, derived from a total of 81 cases. The tongue appears to be the most affected, accounting for 48% (39 cases) of the total tumors. Following this, the oral floor represents 33% (27 cases) of the cases, while the inferior alveolar ridge/gingiva contributes for 19% (15 cases).

Regarding preoperative biopsies, 20 (25%) were positive for PNI and 10 (12%) for LVI.

All the patients underwent a complete removal of the primary OCC with free margins (>5 mm) and to an END.

Regarding the histological analysis, the majority of cases, accounting for 51% (n = 41), were classified as Grade 3 (G3), 39% (n = 32) of patients as Grade 2 (G2), and 10% of patients (n = 8) as Grade 1 (G1).

After surgery, patients were categorized into pathological stages according to AJCC guidelines: pT1 37% (n = 30), pT2 35% (n = 28), pT3 17% (n = 14), and pT4 11% (n = 9).

Patients with coherent clinical and pathological staging were 30/81 pT1 (37%) and 28/81 pT2 (35%), while in 27/81 patients (33%), the clinical staging did not agree with a pathological one.

Regarding the definitive node status, 19/81 (24%) patients resulted positive for lymph node metastases (pN + group), while 62/81 (76%) patients tested negative (pN0 group). During the 24-month follow-up, no patient with N0 lymph node status developed metastases. Among pT1 cases, the identification of two lymph node metastases resulted in two cases being staged as N1. Within the pT2 category, two cases were staged as N1, and an additional case was classified as N2. Among the remaining clinical cases, 14 tumors were designated as pT3, of which 8 exhibited metastasis: 3 N1, 3 N2 (comprising N2b and N2c), and 2 N3c. The final nine cases were staged as pT4, with six of them demonstrating metastasis: two N1, three N2, and one N3.

Regarding the final PNI and LVI, the rates were confirmed (respectively, 25% and 12%).

The patients underwent radio/chemotherapy based on their indications, but none of the 62 pN0 patients developed a neck metastasis in the 24 months of follow-up.

### 3.2. Diagnostic Performance of Inflammatory Biomarkers and Perineural/Lympho-Vascular Invasion

Through the Youden index method, the optimal cut-offs of each individual inflammatory biomarker were calculated to discriminate the patients at higher risk of neck node metastases (PLR: 249.3, NLR: 13.09, MLR: 0.44, SII: 1043.12, and SIRI: 1.85).

Considering these optimal thresholds, an ROC analysis was performed to identify the highest accuracy, sensitivity, and specificity in predicting neck node metastases.

The best accuracy was assessed to be of NLR (81%); the higher sensitivity was recorded to NLR (96%), while the higher sensibility was recorded to SII and SIRI (76%). The best PPV was recorded to NLR (86%), while the higher NPV was assessed to SII (87%).

The best AUCs were performed with NLR and SII (0.73).

**Figure 1:**
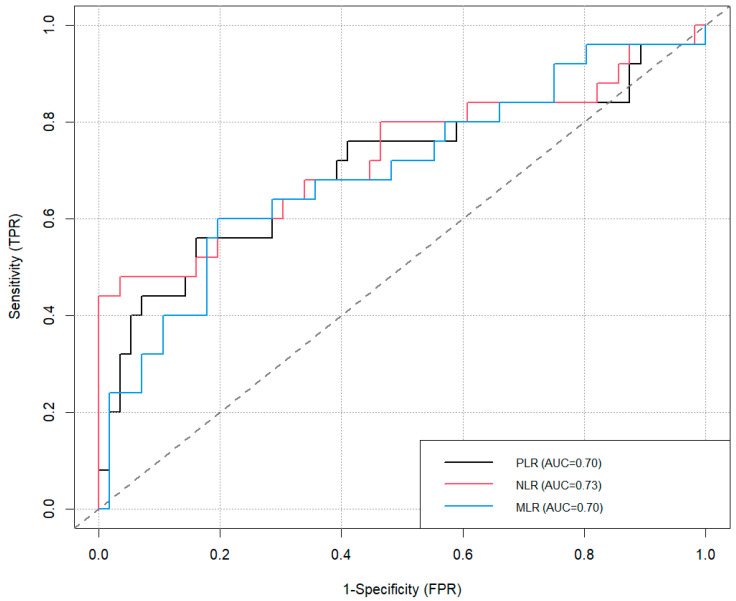
ROC curves of PLR, NLR, and MLR.

**Figure 2:**
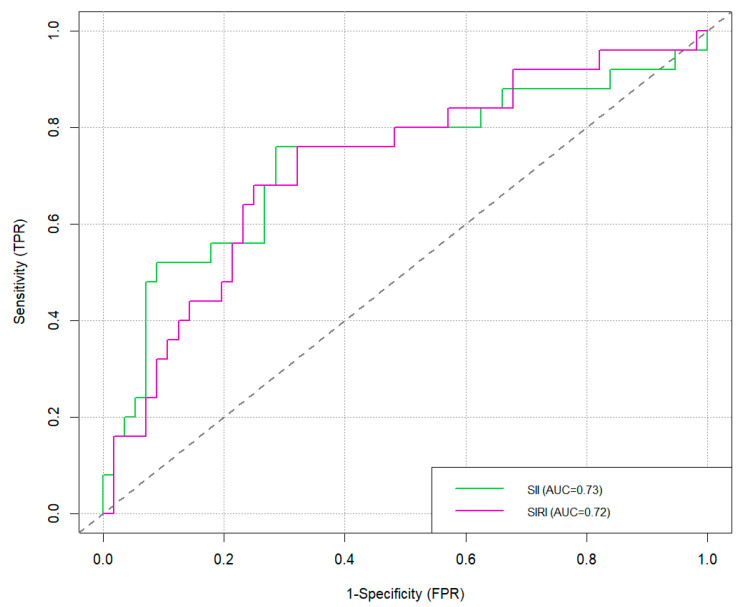
ROC curves of SII and SIRI.

The total values are shown in Table 2 and Figure 1 and Figure 2.

The same analysis was performed to calculate the best accuracy, specificity, sensibility, PPV, and NPV for the PNI and LVI. In particular, the best accuracy was assessed with LVI (83%). Considering that all the patients with LVI showed a pN+, the sensitivity and the NPV of this parameter were recorded to be 100%. The total values for PNI and LVI are shown in Table 3.

### 3.3. Analysis of Main Predictors of Neck Metastases

Univariable logistic regression analysis of the main predictors for higher risk neck node metastases resulted in statistical significance for NLR, PLR, SII, SIRI, and PNI.

The total values are shown in Table 4.

The multivariate analysis was performed considering the statistically significant value, but no further improvement in diagnostic performance was recorded.

## 4. Discussion

Neck metastases represent the most critical prognostic factor in oral cavity carcinoma (OCC), making proper lymph node management essential. While treatment protocols for necks with confirmed metastases are well-established, the approach to clinically negative necks remains controversial. Surgeons currently have several options, including watchful waiting for early-stage tumors versus performing elective neck dissection or sentinel lymph node biopsy.

Key prognostic factors in OCC include tumor differentiation, thickness, depth of invasion, and perineural and lymphovascular invasion, as well as the patient’s inflammatory status [28,29,30]. According to the literature, the prevalence of occult neck metastases may range from 15% to 60%, influenced by these different prognostic factors [31,32].

Growing evidence suggests that inflammation-related biomarkers, including the neutrophil-to-lymphocyte ratio (NLR), lymphocyte-to-monocyte ratio (LMR), platelet-to-lymphocyte ratio (PLR), systemic inflammation response index (SIRI), and systemic immune-inflammation index (SII) are associated with poor prognosis in patients with oral cavity squamous cell carcinoma (OCSCC) [33].

Specifically, in head and neck cancers—such as oral cancer—elevated values of these inflammatory blood markers have been identified as potential indicators of reduced overall survival (OS) and disease-free survival (DFS) [34,35].

The inflammatory microenvironment is a significant factor in cancer progression, with NLR and PLR indicating the balance between inflammatory and regulatory responses. An increased NLR, resulting from higher neutrophil counts or lower lymphocyte levels, highlights this imbalance. Neutrophils commonly accumulate in the tissue surrounding tumors, where they release substantial amounts of vascular endothelial growth factor, creating a favorable environment that supports local tumor invasion and the spread of metastases [36].

Lymphocytes, instead, are key anticancer agents, targeting tumor cells through cytotoxic activity and cytokine production to support the immune response and limit tumor growth. However, in a proinflammatory tumor environment, neutrophils can weaken the lymphocyte-mediated immune response [37].

The SII is based on three parameters, neutrophils, platelets, and lymphocytes, while the SIRI consists of three types of inflammatory cells, neutrophils, monocytes, and lymphocytes [21].

Based on these findings, numerous studies have explored the relationship between inflammatory status and prognosis in patients with oral cell carcinoma (OCC).

Several authors have discussed the role of NLR as a prognostic factor for oral cancer, identifying optimal thresholds above which tumor progression and, therefore, overall survival are worse. Khan et al. [38] identified 3.19 as the optimal cut-off with a specificity of 61%; Werner et al. [39] identified 3.65 with a specificity of 61%; Wu et al. [40] identified 2.95 with a specificity of 68%; and Salem et al. [41] identified 11.5 with a specificity of 69.5%. The analysis of these papers reveals that there is no absolute cut-off, but, at most, it is possible to identify a range, approximately 2.95–11.5, below which there is a low risk of tumor progression, while above which there is a high risk of poor prognosis. In these articles, however, a direct correlation between the values of NLR and the presence of occult metastases is not identified but rather the tumor progression in general. Therefore, our study is more specific regarding this aspect, so much that the specificity recorded for our cutoff is 96%.

The meta-analysis by Yang suggests that both the systemic immune-inflammation index (SII) and the systemic inflammation response index (SIRI) serve as prognostic indicators in oral cancers. Elevated levels of SII and SIRI are associated with poorer overall survival (OS), while high SII also correlates with reduced disease-free survival (DFS) [42].

Neck node metastasis is another important prognostic factor, influenced by many other factors such as DOI, LVI, PNI, pT size, differentiation grade, and margin status. Findings from a meta-analysis by Shuojin et al. have also reported the importance of LVI as a prognostic predictor for metastasis and prognosis in patients with OSCC [15]. Mascitti et al. have also conducted a review on the clinical and prognostic role of LVI in OSCC and confirmed the effectiveness to better define the therapeutic strategies in OSCC patients [13] Recently, several systematic reviews have highlighted the significant prognostic role of PNI in HNSCC. For instance, Binmadi et al. [43] focused on OSCC, while Li et al. [44] focused specifically on tongue squamous cell carcinomas. These studies, along with our own, have collectively validated the predictive value of PNI in survival.

Starting from these premises, our study aimed to demonstrate the direct correlation between the presence of LVI and PNI at preoperative biopsy and high risk of occult neck lymph node metastases in early-stage OSCC with clinically negative neck. Furthermore, our study aimed also to demonstrate that values above specific thresholds of the blood inflammatory markers NLR, PLR, SII, and SIRI are predictors of occult neck lymph node metastases in these patients.

Statistical analyzes conducted using the Youden index method and the calculation of ROC curves revealed the specific cut-offs for each index, above which the risk of metastasis increases: NLR 13.09, PLR 249.30, SII 1043.12, and SIRI 1.85.

NLR was the most accurate test (81%) and was very specific (96%), although it was not very sensitive (48%). On the contrary, SII and SIRI, although very accurate (73%), were very sensitive (76%) and less specific. Univariate regression analysis demonstrated that all markers, except MLR, were statistically significant predictors of neck node metastases.

Therefore, NLR is able to better discriminate the true negatives, while SII and SIRI are better able to discriminate the true positives.

As regards LVI and PNI, both were not very specific but highly sensitive, therefore capable of discriminating true positives. In fact, for example, in our sample, all patients with LVI presented metastases.

These results are very encouraging; in fact, these markers which are easily accessible and reproducible could be routinely evaluated preoperatively for the correct diagnostic classification of the patient with OSCC. Furthermore, the calculation of these markers is very cheap and could be helpful for the economy of hospital companies, which are burdened daily by high costs [45]. The clinical relevance of this study is that these parameters could act as support tools of diagnosis and could help surgeons in the decision-making process, particularly regarding surgical indications for neck lymph node management. In fact, the evaluation of the value of NLR and other biomarkers could be part of a more complete preoperative staging, together with other prognostic factors such as cTNM, perineural and lymphovascular invasion, grading, and age of the patient for the risk stratification and decision of the treatment of the neck lymph nodes (wait and see vs. neck dissection). In this way, it would be possible to avoid overtreatment with unnecessary neck dissection or, on the contrary, an undertreatment with the risk of developing metastases at a later time.

Despite these encouraging premises, our study has some limitations: the small sample size, the retrospective and single-center nature of the analysis, and the possibility of a history of inflammatory diseases, which may create a patient selection bias.

Nevertheless, these preliminary results can be used as a theoretical basis for a prospective multicenter study on a large-scale population to validate this hypothesis. Our future prospect will be to conduct a study also considering innovative artificial intelligence and deep learning techniques, such as radiomics, which will be able to contribute even more to the detection of occult metastases in these patients. Furthermore, it could be interesting to conduct further studies on patients not eligible for elective neck dissection and monitor the changes in their inflammatory indices over time.

## 5. Conclusions

In conclusion, the data obtained from our preliminary study seem to confirm that NLR, PLR, SII, and SIRI are able to detect occult neck node metastases in early-stage OSCC, when above specific threshold values. On the other hand, perineural and lympho-vascular invasion are histological markers highly predictive of occult neck node metastases in these patients. These markers could be used as a diagnostic support tool to preoperatively predict the risk of neck node metastases in these patients and to effectively guide the treatment strategy, although a prospective interventional study is needed to validate these preliminary results.

## Figures and Tables

**Table 1:** Main features of the 81 enrolled patients.

General Population Features	
Variables	Total Cases 81
Age (years)	
<60	35 (43%)
>60	46 (57%)
Gender	
Female	39 (48%)
Male	42 (52%)
Tumor location	
Tongue	39 (48%)
Oral floor	27 (33%)
Inferior alveolar gingiva	15 (19%)
cT status	
cT1	34 (43%)
cT2	47 (57%)
pT status	
pT1	30 (37%)
pT2	28 (35%)
pT3	14 (17%)
pT4	9 (11%)
p Grading	
G1	8 (10%)
G2	32 (39%)
G3	41 (51%)
pN status	
N0	62 (76.5%)
N1	2 (pT1) (2.5%)
	2 (pT2) (2.5%)
	3 (pT3) (3.7%)
	2 (pT4) (2.5%)
N2	
	1 (pT2) (1.2%)
	3 (pT3) (3.7%)
	3 (pT4) (3.7%)
	2 (pT3) (2.5%)
N3	1 (pT4) (1.2%)

Of these patients, 42 (52%) were male, and 39 (48%) were female, with a median age of 64.2 years (interquartile range (IQR): 29–94 years).

**Table 2:** Diagnostic performance of inflammatory biomarkers.

Variable	Optimal Threshold	Accuracy	Specificity	Sensitivity	PPV	NPV
PLR	249.30	75%	84%	56%	61%	81%
NLR	13.09	81%	96%	48%	86%	81%
MLR	0.44	74%	80%	60%	58%	82%
SII	1043.12	73%	71%	76%	54%	87%
SIRI	1.85	70%	68%	76%	51%	86%

**Table 3:** Diagnostic performance of PNI and LVI.

Variable	Accuracy	Specificity	Sensitivity	PPV	NPV
PNI	70%	36%	86%	75%	53%
LVI	83%	44%	100%	80%	100%

**Table 4:** Univariable logistic regression analysis of metastases predictors.

Characteristic	N	OR	95% CI	*p*-Value
**Age**	81	0.9857	0.9519, 1.0203	0.411
**Sex**	81	1.6111	0.6244, 4.2904	0.328
**NLR**	81	1.1724	1.0850, 1.2882	**<0.001**
**PLR**	81	1.0059	1.0024, 1.0100	**0.003**
**MLR**	81	0.9951	0.9923, 1.0032	0.732
**SII**	81	1.0005	1.0002, 1.0008	**0.001**
**SIRI**	81	1.0783	1.0001, 1.0006	**0.001**
**LVI**	81			
NO		—	—	
YES		NE	NE	NE
**PNI**	81			
NO		—	—	
YES		3.3750	1.1160, 10.485	**0.031**

## Data Availability

Data are contained within the article.
